# Magnetometer with nitrogen-vacancy center in a bulk diamond for detecting magnetic nanoparticles in biomedical applications

**DOI:** 10.1038/s41598-020-59064-6

**Published:** 2020-02-12

**Authors:** Akihiro Kuwahata, Takahiro Kitaizumi, Kota Saichi, Takumi Sato, Ryuji Igarashi, Takeshi Ohshima, Yuta Masuyama, Takayuki Iwasaki, Mutsuko Hatano, Fedor Jelezko, Moriaki Kusakabe, Takashi Yatsui, Masaki Sekino

**Affiliations:** 1 Graduate School of Engineering, The University of Tokyo, Tokyo, 113-8656 Japan; 2 National Institutes for Quantum and Radiological Science and Technology (QST), Chiba, 263-8555 Japan; 3 Department of Electrical and Electronic Engineering, Tokyo Institute of Technology, Tokyo, 152-8550 Japan; 4 Institute of Quantum Optics, Ulm University, Ulm, 89081 Germany; 5 Research Center for Food Safety, Graduate School of Agricultural and Life Sciences, The University of Tokyo, Tokyo, 113-8657 Japan; 6 Matrix Cell Research Institute Inc., Ibaraki, 300-1232 Japan

**Keywords:** Sensors and probes, Biomedical engineering

## Abstract

We developed a novel magnetometer that employs negatively charged nitrogen-vacancy (NV^−^) centers in diamond, to detect the magnetic field generated by magnetic nanoparticles (MNPs) for biomedical applications. The compact probe system is integrated into a fiber-optics platform allowing for a compact design. To detect signals from the MNPs effectively, we demonstrated, for the first time, the application of an alternating current (AC) magnetic field generated by the excitation coil of several hundred microteslas for the magnetization of MNPs in diamond quantum sensing. In the lock-in detection system, the minimum detectable AC magnetic field (at a frequency of 1.025 kHz) was approximately 57.6 nT for one second measurement time. We were able to detect the micromolar concentration of MNPs at distances of a few millimeters. These results indicate that the magnetometer with the NV^−^ centers can detect the tiny amounts of MNPs, thereby offering potential for future biomedical applications.

## Introduction

Negatively charged nitrogen-vacancy (NV^−^) color centers in a diamond have been proposed and implemented as a promising magnetic field quantum sensor^1–3^. Optically accessible electron spin states of diamond NV^−^ centers allow initialization and readout of the state. Such accessibility of spin transition allows highly-sensitive detections of magnetic field^4–7^, electric field^8^, temperature^9^, distortion^10^, and quantum computing^11^. This diversity clearly indicates the significant superiority of the method compared with each individual sensing type, and potentially provides a technology to build sensors for a wide range of applications. In particular, magnetic sensing using the diamond NV^−^ center has been explored as one of the most highly sensitive sensors for several decades, potentially providing an order of femtotesla magnetic detection comparable to the sensitivity of the magnetic sensing using the superconducting quantum interference device (SQUID)^12–14^ or optically pumped atomic magnetometer (OPAM)^15–20^. The electron spin of NV^−^ in solid-state material can be controlled at room temperature under an ambient magnetic field (for example, a geomagnetic field of approximately 50 μT). In contrast, SQUID and OPAM require a refrigerant with liquid nitrogen/helium to retain the superconducting conditions and ambient low magnetic field (less than several tens of nanoteslas) to satisfy the spin exchange relaxation-free condition^17^, respectively, to realize the highly sensitive magnetic detection. Moreover, the diamond NV center covers a wide range of sensing volumes, from the nanometer to the millimeter scale. A single NV^−^ center in a nano-diamond enables the detection of the phenomena in the order of the nanometer scale, such as the magnetic field generated by a cell in biomedical tissues^21,22^. An ensemble of the NV^−^ center in a bulk diamond can observe the phenomena in the order of the millimeter scale^23^, which is one of the unique features of diamond quantum sensing. Furthermore, the four possible orientations of the NV^−^ axis in a bulk diamond enable demonstrations of vector imaging^24–26^.

As an application of this effective quantum magnetic sensing, in this study, we investigated the detection of magnetic nanoparticles (MNPs) in biomedical tissues. Thus far, MNPs have been employed extensively in biomedical applications, such as the diagnosis and therapy of various diseases^27–29^, because the magnetic technique using MNPs provides minimally invasive treatment and diagnosis, compared with the conventional approaches using radiation or radioactive particles^30^. For example, the detection of MNPs accumulating into sentinel lymph nodes is useful for the diagnosis of cancer metastasis in breast cancer patients. Recent advanced clinical/engineering studies for breast cancer patients have described the detection of MNPs using a magnetometer with a conventional magnetic sensor, such as a Hall sensor or pickup coil, as a promising technique that offers an alternative to harmful radioactive particles^31–37^. The detectable distance is approximately 10 mm for 140 μg of MNPs. It is necessary to develop highly sensitive magnetic detection to detect MNPs that are deeper inside the biomedical tissue, and create a new and applicable clinical regime.

In this study, we developed a novel magnetometer with NV^−^ centers in a bulk diamond, and demonstrated the detection of the magnetic field from MNPs. The optical fiber-based system enabled us to realize a compact probe system as opposed to a confocal-based optical system, and alternating-current (AC) magnetic fields of the excitation coil system facilitated highly sensitive detection of MNPs.

## Results

### NV^−^ center in bulk diamond and lock-in detection of alternating magnetic field

**Figure 1:**
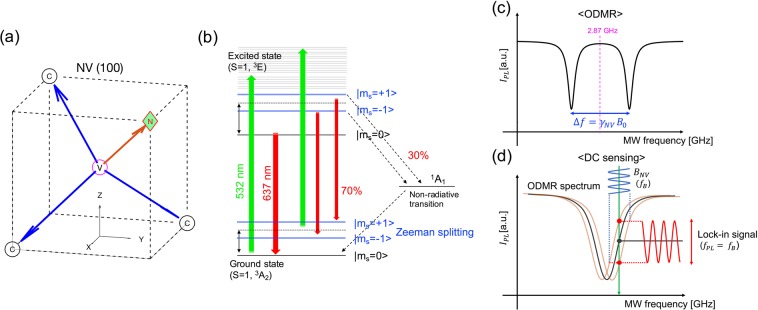
(**a**) Schematics of NV (100) lattice in diamond. N, C, and V represent the nitrogen atom, carbon atom, and vacancy, respectively. The angle between any two arrows is 109.5° in a regular tetrahedron structure in diamond. (**b**) Energy level of NV^−^ center in diamond. The NV^−^ is excited by the green laser and then emits red fluorescence of the NV according to the electron energy states. An energy level of *m*_*s*_ = ±1 excited by MW (*f*_*ESR*_ ~2.87 GHz in the case of zero magnetic fields) increases to excited states and relaxes via non-radiative transition to ground states. Moreover, *m*_*s*_ = ±1 are split by external magnetic fields owing to the Zeeman effect. (**c**) ODMR under an external magnetic field. (**d**) Principle of lock-in detection for AC magnetic field sensing at certain MW frequency.

We used a (100) diamond bulk (2 × 2 × 0.5 cm^3^) irradiated with high-energy electrons. Figure 1(a) presents the structure of the NV-axis and three C-axes of the (100) diamond in the Cartesian coordinate system; the axes are 109.5° apart. The NV^−^ center is randomly arranged along the crystallographic orientation of the diamond lattice, leading to the existence of four possible orientations of the NV^−^ center in the diamond. The principle of magnetic sensing is based on the energy level structure of the electrons, as illustrated in Fig. 1(b). The energy state of the NV^−^ center that possesses an electron spin can be controlled by a green laser (532 nm) and microwave (MW) irradiation, resulting in a decrease in the red fluorescence intensity attributed to the electron spin resonance (ESR). Under an external magnetic field, the energy level of *m*_*s*_ = ±1 splits according to the magnetic field strength owing to the Zeeman effect, which also results in the shifting of the red fluorescence in the optically detected magnetic resonance (ODMR) spectrum (Fig. 1(c)). The magnitude of the shift Δ*f* from the center frequency *f*_0_ (approximately 2.87 GHz without the magnetic field) can be expressed by

1 $$\Delta f={\gamma }_{NV}{B}_{0},$$

where *γ*_*NV*_ = *μ*_*B*_g_*s*_/*h*~28.024 GHz/T is the gyromagnetic ratio, *μ*_*B*_ is the Bohr magneton, *g*_*s*_ ≈ 2 is the Landé factor, *h* is the Planck constant, and *B*_0_ is the applied magnetic field strength along an NV^−^ axis. Figure 1(d) illustrates the principle of the lock-in detection (known as DC sensing) that we employed for the AC magnetic field sensing. When applying AC magnetic fields under a certain MW frequency, the red fluorescence intensity in a dip also fluctuates according to the strength of the AC magnetic fields. This fluctuation of the red fluorescence signal represents the alternation of the magnetic field strength.

### Development of an optical fiber-based system of a magnetometer using NV^−^ in bulk diamond

**Figure 2:**
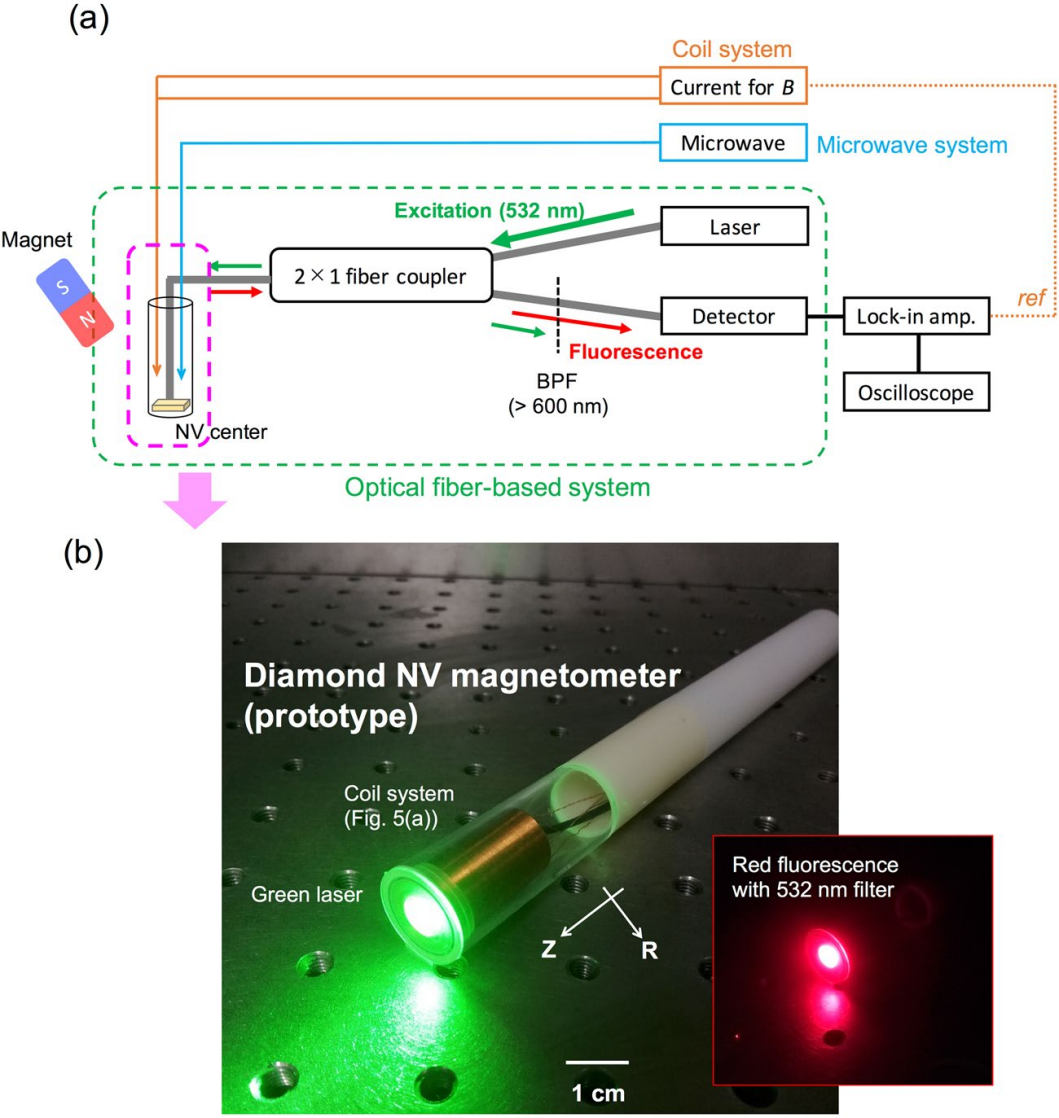
(**a**) Schematic of driving and lock-in detection system of a magnetometer with diamond NV^−^ center: MW for generating ESR, green laser for excitation of electron energy state of NV^−^ center, photodetector for measuring red fluorescence, and electric current for generating AC magnetic fields. Excitation and luminescence light enters and returns through a bifurcated optical fiber bundle, respectively. The external magnetic fields of a permanent magnet produce dip splitting of each NV axis. (**b**) Image of the developed diamond NV magnetometer (prototype). The green laser excites red fluorescence in the diamond NV. The longitudinal and lateral directions are the *Z*-axis and *R*-axis, respectively.

A schematic overview of a magnetometer with an optical fiber-based system is illustrated in Fig. 2(a). A green laser (532 nm: frequency doubled YAG laser) excited the NV^−^ center through the fiber port of a 2 × 1 fiber coupler. The red fluorescence originating from the excited NV^−^ center was detected by a photodiode (PD) through a longpass filter (>600 nm) and the same fiber port of the fiber coupler. MW (50 dB) was applied to the NV^−^ center by using a thin copper film (approximately 0.04 mm) located beneath the bulk diamond surface. AC magnetic fields (sinusoidal waves) for magnetization of the MNPs were generated by a coil system consisting of an excitation and a cancellation coil. The variation in the detected red fluorescence was measured using the lock-in amplifier and oscilloscope, representing the lock-in detection system. A permanent magnet located close to the probe head produced eight dips of red fluorescence. Figure 2(b) presents an image of the handheld part of the prototype magnetometer, with a green light for the NV^−^ excitation and red light generated by the excited NV^−^ center. The incident laser power to the bulk diamond was approximately 100 mW.

**Figure 3:**
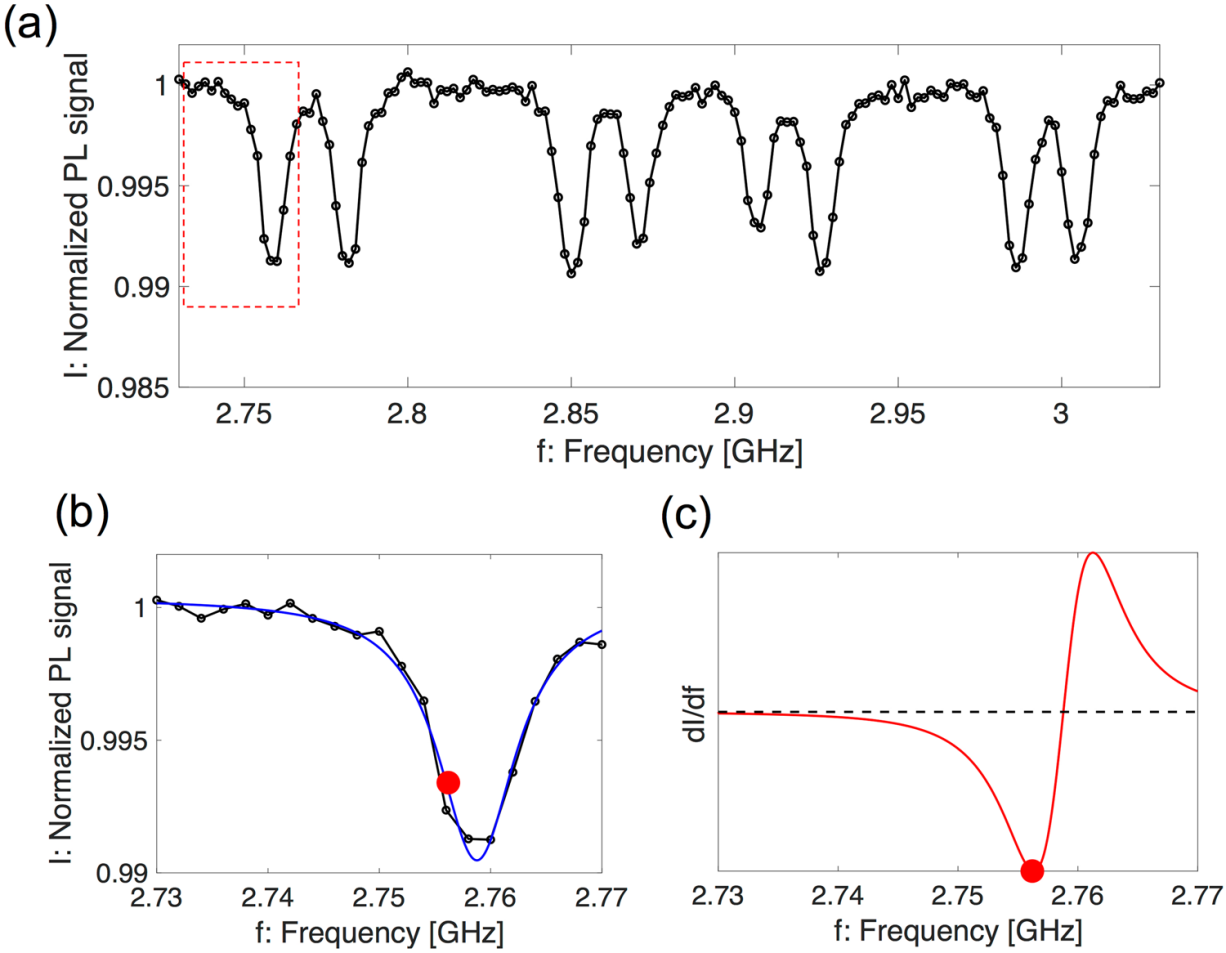
CW–ODMR spectrum: red luminescence as a function of MW frequency. (**a**) Under the static magnetic field of a permanent magnet, four NV orientations produce eight dips based on the Zeeman effect. (**b**) Enlarged view of the dip with the lowest frequency (dotted square region in (**a**)). The blue line represents the curve fitted by the Lorentz function (Eq. (2)). (**c**) The signal $\frac{dI(f)}{df}$ can be detected by the lock-in amplifier.

The permanent magnet played an important role in applying a constant magnetic field to extract one axis from four NV-axes in the lock-in detection. It was clearly demonstrated that the four NV orientations produced eight dips (four-pair splitting) under a static magnetic field in the continuous wave (CW)–ODMR spectrum, according to Eq. (1), as shown in Fig. 3(a). We selectively utilized the dip with the lowest frequency for sensing the AC magnetic fields (Fig. 3(b)). The shape of the measured fluorescence intensity *I*(*f*) of the dip can be fitted by the following Lorentzian profile:

2 $$I(f)=1-C\frac{{\omega }^{2}}{{(f-{f}_{0})}^{2}+{\omega }^{2}},$$

where *C* is the dip depth, *f*_0_ is the dip center frequency, and *ω* is the half width at half maximum (HWHM) of the dip. The dip depth (contrast) was approximately 1%, and the HWHM of the dip was approximately 4.3 MHz. The steepness of the dip, $|\frac{dI(f)}{df}|$, represents the magnetic sensitivity in the lock-in detection. We selected the frequency at the highest $|\frac{dI(f)}{df}|$ to generate the largest signal for the lock-in detection. The maximum signal was detected at *f* ~2.7562 GHz, where the slope of the ODMR spectrum was steep, as shown in Fig. 3(c). According to the dip profiles, each NV axis received static magnetic fields in the range of 0.7–4.4 mT of the permanent magnet.

### Magnetic sensitivity of developed prototype magnetometer

**Figure 4:**
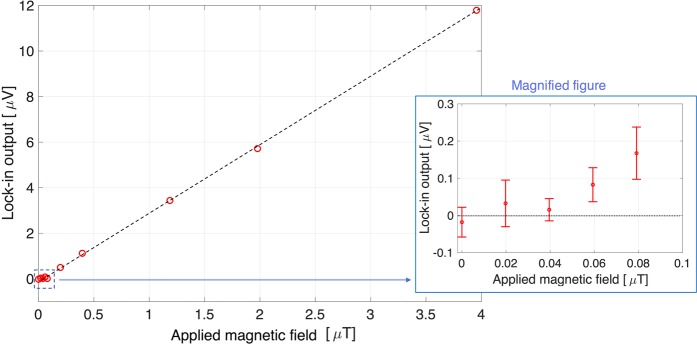
Magnetic sensitivity for external AC magnetic fields *B*_*z*_ with frequency of 1.025 kHz generated by a circular coil in the lock-in detection. The detectable magnetic field strength is approximately 57.6 nT, and linearly proportional to the external AC magnetic field strength. Considering the angle between the NV^−^ center and the magnetic field *B*_*z*_, the sensitivity is approximately 33.2 nT. Error bar represents a standard deviation of measured data points.

The magnetic sensitivity of the prototype magnetometer using the NV^−^ center was evaluated under the application of external uniform AC (1.025 kHz, without the influence of the harmonics of 50 Hz commercial frequency) magnetic fields generated using a circular coil (30-mm radius and 2 turns). Figure 4 shows the output voltage detected by the lock-in detection system, under various AC magnetic field strengths. The minimum detectable magnetic field was approximately 57.6 nT (with a signal-to-noise ratio of approximately 1) with time-averaging of 1 s as the magnetometer sensitivity. Considering the angle of the NV^−^ center, the best sensitivity, as the sensitivity of NV^−^ center, was approximately 33.2 nT: 57.6 × cos109.5°/2.

### Development of coil system of a magnetometer and detection of MNPs

**Figure 5:**
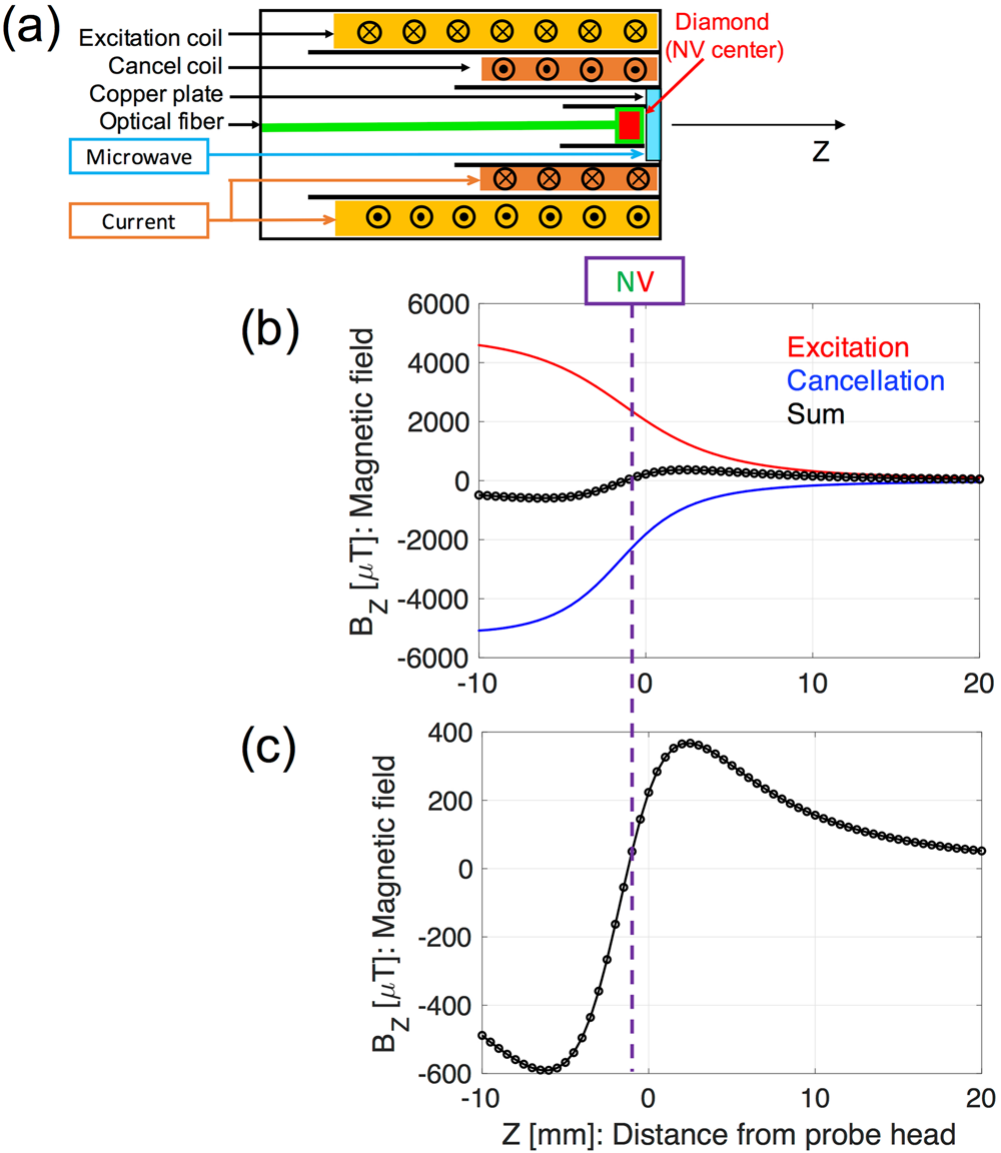
(**a**) Structure of inside of magnetometer head: excitation coil, cancellation coil, MW coil, diamond NV, and optical fiber. (**b**) Calculated magnetic field of excitation (red) and cancellation (blue) coils on *Z*-axis. The black line indicates the summation of the excitation and cancellation magnetic fields. (**c**) Enlarged figure for only resultant magnetic fields.

For effective detection of the magnetic field from the MNPs (Resovist®^38^), we used the excitation coil inside the magnetometer with the bulk diamond. The application of the AC magnetic fields yielded AC magnetization of the MNPs, and magnetic sensing of the NV^−^ center could detect newly generated magnetic fields of the magnetized MNPs. Obviously, larger applied magnetic fields lead to greater magnetization of the MNPs, resulting in relatively easier detection compared with that without excitations. However, when applying the AC magnetic fields, the NV^−^ center in the diamond also received excitation magnetic fields, creating difficulties in the MNPs detection, because the magnetic fields of the MNPs (in the order of sub-microtesla) were substantially smaller than those of the excitation coil (in the order of sub-millitesla). Therefore, the cancellation coil played an important role in eliminating the excitation magnetic field at the location of the active NV^−^ center area. Figure 5(a) shows the components of the magnetometer head. The diamond with the NV^−^ center was located at the center of the magnetometer head, at the 0.5 mm on the inner side from the probe head surface. A MW coil, a cancellation coil, and an excitation coil were arranged from the inside to the outside of the magnetometer. The outer diameter of the probe head was 18 mm. The detailed specifications of the excitation and cancellation coils are summarized in Table 1. Figure 5(b) shows the magnetic field distributions *B*_*z*_(*Z*) calculated using the numerical simulations based on the Biot–Savart law of the excitation and cancellation coils. The probe head surface was located at *Z* = 0 mm and the bulk diamond location was *Z* = −0.5 mm. At the diamond location, although the excitation coil produced approximately 2600 μT, the resultant magnetic field was reduced to 1 μT under the application of the cancellation coil (Fig. 5(c)). In the experiment, the residual magnetic field strength could be estimated by the output voltage as a function of the applied magnetic fields (Fig. 4). The determined value was 3.5 μT, which indicates that approximately 99% of the excitation magnetic field was canceled out at the location of NV^−^ center. At the front of the probe head (*Z* = 0–10 mm), the resultant magnetic field was 150–360 μT for the MNP magnetization.

**Table 1:** Detailed structures of excitation and cancellation coils.

Coil	Outer/inner diameter [mm]	Wire ϕ [mm]	Length [mm]	Turns	R [Ω]	L [mH]
Excitation	15.4/11	0.3	28.8	800 (8 × 100)	10.9	2.7
Cancellation	10.2/8	0.18	19.8	588 (6 × 98)	11.9	1.1

**Figure 6:**
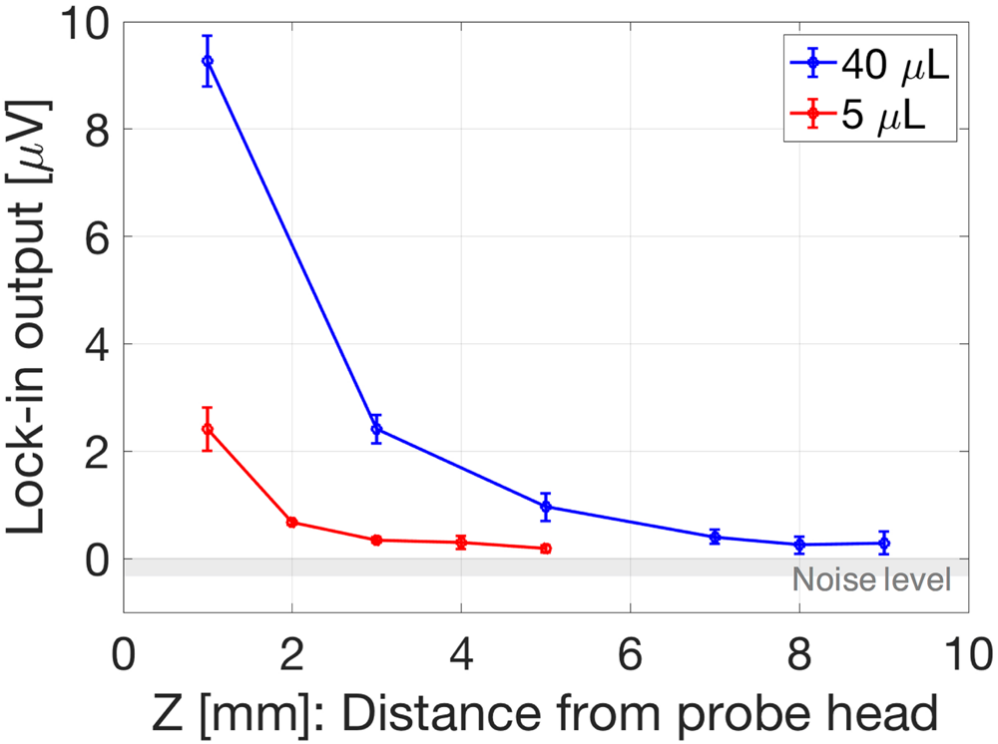
Measured voltage with respect to 40 μL (1120 μg: blue solid line) and 5 μL (140 μg: red solid line) of MNPs. The longitudinal detectable distances of 40 μL and 5 μL were 9 and 5 mm, respectively. The experimentally measured noise level (the standard deviation of measure data points: gray-color area) was approximately 400 nV.

Figure 6 shows the signals that originated from the MNPs using the developed magnetometer. The background signal which is attributed to the residual magnetic fields was subtracted from these signals. The strength of the detected signal decreased upon increasing the distance from the probe head. The longitudinal detectable distances of the MNPs of 40 μL (1120 μg) and 5 μL (140 μg) were 9 and 5 mm, respectively, and the MNPs could be detected successfully by using the newly developed magnetometer with NV^−^ centers in the bulk diamond.

## Discussion

We demonstrated the detection of MNPs using the magnetic sensing of NV^−^ centers in a bulk diamond installed inside a handheld magnetometer, for biomedical applications. Although magnetic quantum sensing with NV^−^ centers potentially possesses a sub-picotesla detection ability^6,7^, the detected magnetic sensitivity was 57.6 nT. The shot-noise limited magnetic field sensitivity *η* is derived by the following equation, based on the ODRM spectrum (Fig. 3(b))^4^:

3 $${\rm{\eta }}\sim \frac{1}{{\gamma }_{NV}}\frac{2\omega }{C\sqrt{{I}_{0}}},$$

where *I*_0_ [cps] is the photon count of the red luminescence per second. According to the signal measured by the PD, the estimated red luminescence power was 3.6 μW, indicating that *I*_0_ was approximately 1.2 × 10^13^ [cps]. Therefore, the estimated theoretical magnetic sensitivity *η* was approximately 9 nT, which is higher than the experimental sensitivity (33.2 nT with respect to the NV^−^ center, as shown in Fig. 4). The electric noise of several hundred nanovolts may have limited the experimental sensitivity. In addition, the noise level without applying the lock-in technique was approximately 57 μV (the noise level with the lock-in technique applied was approximately 400 nV), indicating that the lock-in detection technique enhanced the sensitivity up to 140 times; the noise level represents the sensitivity limit considering that the signal to noise ratio is 1.

When an external magnetic field is applied to the volume containing MNPs, the magnetic fields generated by the magnetized MNPs, *B*_*MNP*_, is calculated using the following equation:

4 $${B}_{MNP}=\frac{{{\rm{\mu }}}_{0}}{4{\rm{\pi }}}(-\frac{{\boldsymbol{m}}}{{|{\boldsymbol{r}}|}^{3}}+\frac{3({\boldsymbol{m}}\cdot {\boldsymbol{r}}){\boldsymbol{r}}}{{|{\boldsymbol{r}}|}^{5}}),$$

where μ_0_ is the vacuum magnetic permeability, ***m*** [A·m^2^] is the magnetic moment of the MNPs revealed by the SQUID measurement^36^, and *r* [m] is the distance between the MNPs and NV^−^ center. The numerical *B*_*MNP*_ values with respect to 40 and 5 μL were 60.2 and 84.9 nT, respectively, which also correspond to the experimental sensitivity (Fig. 4). We believe that this study provides a pioneering method for detecting the MNPs actively magnetized by excitation magnetic fields.

As mentioned previously, larger residual magnetic fields at the NV^−^ center location interrupted the magnetic sensing of the MNPs, indicating the requirement for a small residual magnetic field for achieving highly sensitive magnetic detection of the MNPs. The large residual magnetic field distorted the waveform in the lock-in detection, leading to a reduction in the magnetic signal with the fundamental frequency of the MNPs, owing to the generation of the harmonics. In the MNP-detection experiment, the strength of the residual magnetic fields was approximately 3.5 μT, according to the sensitivity depicted in Fig. 4. For this actual condition, our numerical calculation demonstrated that this residual magnetic field is sufficiently small to maintain the magnetic sensitivity with respect to the MNP detection (Fig. 6). The result indicates that the coil system with excitation and cancellation coils is useful for the effective detection of MNPs.

We consider that there are two possible means of enhancing the magnetic sensitivity. The first method would be to increase the red luminescence intensity^39,40^. As indicated in Eq. (3), the minimal magnetic sensitivity *η* is inversely proportional to the square root of the intensity. In the case of a hundredfold *I*_0_, the magnetic sensitivity increases up to approximately 0.9 nT, indicating that the detectable distance could reach >10 mm, resulting in the application of the MNP detection in clinical trials for breast cancer patients^35,36^. The second method would be to optimize the coil system for magnetizing the MNPs. A larger excitation magnetic field produces larger magnetic moments, resulting in a larger magnetic signal originating from the MNPs (according to Eq. (4)). While the residual magnetic field at the NV^−^ center was significantly suppressed by the cancellation coil, the excitation magnetic field for magnetizing the MNPs at a distance of 5–20 mm was reduced to approximately 50%. In the near future, we can develop an optimized coil system that can produce larger excitation fields for the MNPs while maintaining small residual magnetic fields at the location of the NV^−^ center. By enhancing the photon signal and excitation of the magnetic field, we will obtain a detectable distance of 20 mm for MNPs located at deeper inside the biomedical tissues.

In conclusion, we have demonstrated a prototype of the novel magnetometer with NV^−^ centers in a bulk diamond, and the detection of MNPs. The optical fiber-based system provided a more compact probe system compared with the confocal-based optical system, and the application of the AC magnetic fields generated by an excitation coil system yielded highly sensitive detection of the MNPs. The AC magnetic sensitivity was approximately 57.6 nT in the lock-in detection system. The sensitivity with respect to the MNP detection using the developed magnetometer was strongly dependent on the residual excitation fields. To retain this magnetic sensitivity, we used a cancellation coil to eliminate the residual magnetic fields at the NV^−^ center location. Consequently, we achieved detectable distances of 9 and 5 mm for the MNPs of 40 μL (1120 μg) and 5 μL (140 μg), respectively. These results indicate that the developed magnetometer can detect the tiny amounts of MNPs. For further improvements, we will optimize the coil system for strong magnetization of MNPs and enhance the red luminescence extraction efficiency, which could lead to highly sensitive detection.

## Material and Methods

### Fabrication of NV^−^ center in diamond

The scale of the bulk diamond (Sumitomo Electric Industries, Ltd., Japan) is 2 × 2 × 0.5 mm^3^. The fabrication process comprised high-energy electron beam irradiation (4.6 MeV at a dose of 1 × 10^18^ cm^−2^) and subsequent thermal annealing (1 hour at 800 °C).

### Optical fiber-based system

**Figure 7:**
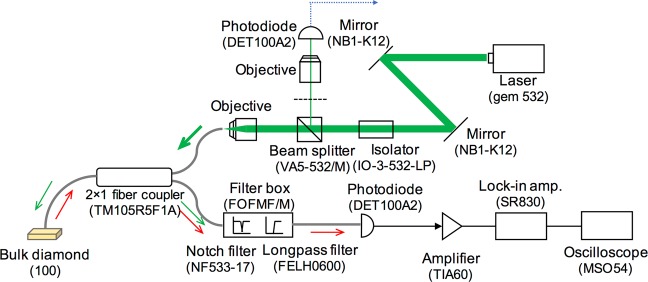
Detailed optical fiber-based system.

The optical fiber-based system is presented in Fig. 7. A green laser (gem 532, 250 mW, Laser Quantum, UK) was injected into the bulk diamond through an isolator (IO-3-532-LP, Thorlabs, USA) a beam splitter (VA5–532/M, Thorlabs, USA), and a 2 × 1 fiber coupler (TM105R5F1A, Thorlabs, USA: 105 μm core diameter, 50:50 split). The red luminescence from the diamond was measured using a photodiode (DET100A2, Thorlabs, USA) through a bifurcated optical fiber bundle, the longpass filter (FELH0600, Thorlabs, USA: >600 nm) and notch filter (NF533-17, Thorlabs, USA: 533 nm) in a filter box (FOFMF/M, Thorlabs, USA). The detected luminescence was measured using a lock-in amplifier (SR830, Stanford Research Systems, USA) through a transimpedance amplifier (TIA60, Thorlabs, USA). A MW generator (MG3740A, Anritsu, Japan) and amplifier (ZHL-16W-43-S+, +45 dB, Mini-circuits, USA) were applied to the NV via a copper thin plate (the width and length were approximately 5 mm, and the thickness was 0.04 mm). The bulk diamond and the edge of the optical fiber were connected using an optical adhesive (Norland Optical Adhesive 68, Norland Products Inc., USA) that was cured under ultraviolet light exposure. The core diameter of the fiber was 105 μm (NA = 0.22), and the estimated active area of the diamond was approximately 100 μm × 100 μm × 500 μm.

### Coil system, power supply, and permanent magnet

The solenoid-shaped excitation and cancellation coils were manufactured by Nihon Universal Electric Co., Ltd, Japan. The electric current (160 mA) produced by the function generator (WF1948, NF Corporation, Japan) was amplified by the power amplifier (HSA4052, NF Corporation, Japan). The MW coil, cancellation coil, and excitation coil were housed from the inner to the outer parts inside the probe head. A column-shaped permanent magnet (Neodymium, ND0848, NdFeB, manufactured by Magfine Corporation, Japan) with a diameter of 25 mm and length of 30 mm, was located close to the bulk diamond, to create eight dips in the ODMR.

### Detection of magnetic nanoparticles (Resovist®; magnetic fluid containing MNPs)

The MNPs used in this study were commercially available Resovist®^38^ (Kyowa CritiCare Co., Ltd., Japan), originally developed for a contrast agent on magnetic resonance imaging (MRI). The MNPs are superparamagnetic iron oxide nanoparticles (SPIONs), with a hydrodynamic diameter of approximately 60 nm. The iron concentration of the magnetic fluid containing MNPs is approximately 28 mg/mL. The magnetic fluids were filled in an acrylic container, for the MNP-detection experiment.
